# Ventricular Arrhythmias and Sudden Death Following Percutaneous Pulmonary Valve Implantation in Pediatric Patients

**DOI:** 10.1007/s00246-022-02881-5

**Published:** 2022-04-08

**Authors:** Pierre-Olivier Veillette, Joaquim Miro, Paul Khairy, Sylvia Abadir, Mathieu Le Bloa

**Affiliations:** 1 Department of Pediatrics, CHU Mère-Enfants Sainte-Justine, Université de Montréal, Montreal, QC Canada; 2 Division of Cardiology, Department of Pediatrics, CHU Mère-Enfants Sainte-Justine, Université de Montréal, Montreal, QC Canada; 3 Electrophysiology Service and Adult Congenital Heart Center, Montreal Heart Institute, Université de Montréal, Montreal, QC Canada; 4 Division of Cardiology, CHUV, Rue du Bugnon 46, 1011 Lausanne, Switzerland

**Keywords:** Congenital heart disease, Ventricular arrhythmia, Tetralogy of Fallot, Pulmonary valve replacement, Pediatrics, Children

## Abstract

Reports have suggested a transient increase in ventricular ectopy early after percutaneous pulmonary valve implantation (PPVI). Little is known about the potential for more serious ventricular arrhythmias (VA) in children who undergo PPVI. We sought to evaluate the incidence of severe VA following PPVI in a pediatric population and to explore potential predictive factors. A retrospective cohort study was conducted of patients who underwent PPVI under 20 years of age in our institution from January 2007 to December 2019. The primary outcome of severe VA was defined as sustained and/or hemodynamically unstable ventricular tachycardia (VT), inducible sustained VT, or sudden death of presumed arrhythmic etiology. A total of 21 patients (mean age 16.2 ± 2.1 years; 66.7% male) underwent PPVI. The majority of patients (*N* = 15; 71.4%) had tetralogy of Fallot (TOF) or TOF-like physiology, with the most common indication being pulmonary insufficiency (*N* = 10; 47.6%). During a median follow-up of 29.6 months (IQR 10.9–44.0), severe VA occurred in 3 (14.3%) patients aged 15.6 (IQR 14.7–16.1) a median of 12.3 months (IQR 11.2–22.3) after PPVI. All events occurred in patients with TOF-like physiology following Melody valve implant. In conclusion, severe VA can occur long after PPVI in a pediatric population, particularly in those with TOF-like physiology. Further studies are required to elucidate underlying mechanisms and assess strategies to mitigate risks.

## Introduction

Pulmonary valve replacement (PVR) for severe pulmonary regurgitation and/or stenosis is often required in patients with congenital heart defects (CHD), most often those with tetralogy of Fallot (TOF). Indeed, while initial surgical correction for TOF allows the majority of patients to survive well into adulthood [1], it is often associated later in life with the need for PVR.

First described in September 2000, percutaneous pulmonary valve implantation (PPVI) offers an attractive alternative to surgical pulmonary valve replacement (sPVR) [2]. Percutaneous valves have demonstrated beneficial hemodynamic effects and a seemingly favorable short- and medium-term safety profile in both adult [3–9] and pediatric cohorts [10, 11]. However, some series reported an increase in the incidence of premature ventricular contractions (PVC) and non-sustained ventricular tachycardia (NSVT) immediately after the procedure, which mostly resolved by 6 months of follow-up [12–14]. While these reports may be a cause for concern, the association between PPVI and more severe ventricular arrhythmias (VA) in children and adults is less clear. The purpose of this study was to assess the incidence of severe VA following PPVI in a pediatric population and to explore potential predictive factors.

## Methods

### Study Population and Baseline Characteristics

A retrospective cohort study was conducted on all patients < 20 years of age who underwent PPVI at the CHU Sainte-Justine from January 2007 (date of the first implant at our institution) to December 2019. All patients had a diagnosis of CHD and met established indications for PPVI, namely significant pulmonary regurgitation and/or stenosis of the native pulmonary valve or of the right ventricle to the pulmonary artery conduit (RV-to-PA conduit) resulting in significant RV dilatation and/or LV or RV dysfunction, with or without associated symptoms [15, 16].

Data were collected on demographic and clinical characteristics, including underlying type of CHD, surgical history, and all interventions prior to PPVI. In addition, preimplantation electrophysiological data [ECG, 24-h Holter, exercise ECG testing and electrophysiology study (EPS)] and imaging metrics [transthoracic echocardiogram, cardiac computed tomography (CT) scan and cardiac magnetic resonance imaging (CMR)] were extracted. Follow-up data, including from subsequent electrophysiological and imaging studies, were collected at 15–30 days post-PPVI, at 1–3 months and at the last clinic visit at our institution (prior to transferring to an adult institution). Each ECG was reviewed to assess the PR interval, QRS duration (QRSd), and corrected QT (QTc) calculated manually using Bazett’s formula. Each EPS was performed under local anesthesia and conscious sedation, using continuous infusion of propofol and bolus of ketamine if required. Programmed electrical stimulation was conducted associated with 3-dimensional electroanatomic mapping of the RV, allowing velocity assessment of potential critical isthmus. The study protocol was approved by our institution’s research ethics committee.

### Percutaneous Pulmonary Valve Implantation

**Fig. 1:**
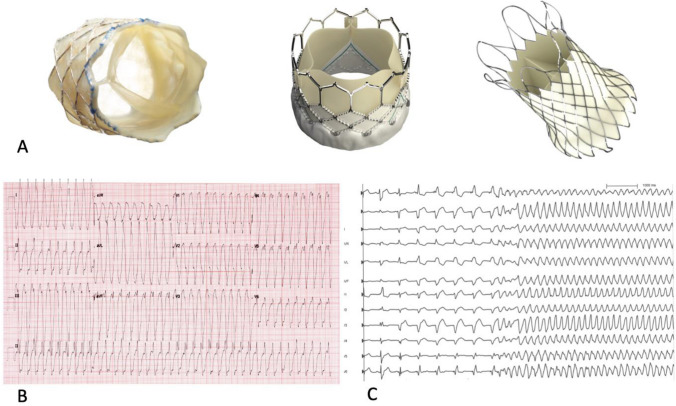
**A** Three models of percutaneous valve implanted in the pulmonary position in our population: the Melody® valve (left panel, reproduced with permission from Medtronic Inc), the Edwards Sapien® valve (middle panel, reproduced with permission from Edwards Inc) and the Venus P-valve® (right panel, reproduced with permission from Medtech). **B** 12-lead ECG recorded in patient #1 who presented to the emergency department for sustained palpitations and demonstrating spontaneous monomorphic ventricular tachycardia (cycle length 250 ms). **C** 12-lead ECG recorded in patient #2 during programmed ventricular stimulation. A rapid sustained monomorphic ventricular tachycardia (cycle length 200 ms) was induced during apical right ventricular pacing at a drive train of 600 ms with three extra-stimuli

Data regarding the type and size of the pulmonary valve and hemodynamic measurements were tabulated. Available percutaneous valves at our institution were the Melody® valve (Medtronic Inc, Minneapolis, MN), the Edwards Sapien® valve (Edwards Lifesciences LLC, Irvine, CA) and the Venus P-valve® (Venus MedTech, Shanghai, China) (Fig. 1A). Information on major intraoperative adverse events were collected (i.e., coronary compression, tamponade, and major bleeding requiring medical/or surgical intervention).

### Outcomes and Follow-Up

The primary outcome was severe ventricular arrhythmia (VA) during follow-up, defined as sustained (lasting 30 s or more) and/or hemodynamically unstable ventricular tachycardia (VT), inducible sustained VT during an EPS, or presumed sudden arrhythmic death, whether aborted or not. Information was also collected on non-arrhythmic deaths, major and minor extracardiac events.

### Statistical Analysis

Non-continuous data are presented as frequency with percentages. Continuous normally distributed variables are presented as mean ± standard deviations and non-normally distributed variables as median and interquartile range (IQR). To study the effect of PPVI on ECG and echocardiographic parameters, related continuous variables were compared using the Wilcoxon signed rank test. A *p* value < 0.05 was considered statistically significant. Statistical analysis was performed using SPSS statistics 26.0 (IBM Corp, Armonk, NY).

## Results

### Patient Characteristics and Procedural Data

A total of 21 patients [14 males (66.7%)], underwent PPVI during the study period, all of whom were included in the study. Patient characteristics are listed in Table 1. Mean age at the time of implant was 16.2 ± 2.1 years with a mean BMI of 20.0 ± 3.2 kg/m^2^. A total of 15 (71.4%) of patients had TOF (*N* = 11; 52.4%) or TOF-like physiology (*N* = 4; 19.1%) (see Table 1). Corrective surgery was performed at a median age of 13.3 (IQR 8.6–24.7) months.

**Table 1:** Baseline patients’ characteristics

Total cohort	*N* = 21
Sex male, *n* (%)	14 (66.7)
Mean age at PPVI, years (SD)	16.2 (± 2.1)
Mean weight at PPVI, kg (SD)	54.4 (± 11.7)
Mean BMI at PPVI, kg/m^2^ (SD)	20.0 (± 3.2)
Congenital anatomic diagnosis, *n* (%)	
Tetralogy of Fallot	11 (52.4)
TOF-like physiology	4 (19.1)
Congenitally corrected transposition of the great arteries with pulmonary atresia and VSD	2 (9.5)
Double-outlet right ventricle	1 (4.8)
VSD with pulmonary stenosis	1 (4.8)
Left obstructive heart disease (s/p ROSS procedure)	3 (14.2)
Truncus arteriosus	2 (9.5)
Isolated valvular pulmonary stenosis	1 (4.8)
Clinical status prior to PPVI	
NYHA status, *n* (%)	
1	7 (33.3)
2	12 (57.2)
3	2 (9.5)
4	0
History of palpitations, *n* (%)	2 (9.5)
History of syncope, *n*	0
Prior surgeries	
Median number of previous surgeries, *n* (IQR)	2 (1–3)
Median age at corrective surgery, months (IQR)	13.3 (8.6–24.7)
Primary indication for implantation, *n* (%)	
Stenosis	1 (4.8)
Regurgitation	10 (47.6)
Mixed lesion (stenosis and regurgitation)	10 (47.6)

*PPVI* percutaneous pulmonary valve implantation, *BMI* body mass index, *TOF* tetralogy of Fallot, *VSD* ventricular septal defect

Pre-implant, 7 patients (33.3%) were in NYHA class I, 12 (57.2%) were in NYHA class II, 2 (9.5%) were in NYHA class III. No patient was in NYHA class IV. Also, 2/21 patients (9.5%) reported palpitations, and all denied syncope. Electrocardiographic and echocardiographic data before and after PPVI are summarized in Table 2. Before implant, the average QRSd was 155 ± 28 ms, with 5 patients (23.8%) having a QRSd greater than 180 ms. In addition, 15 patients (71.4%) had an exercise testing and 12 patients (57.1%) had 24-h Holter monitoring prior to PPVI. No significant arrhythmias were recorded. Fourteen patients (14/21, 66.6%) had a pre-PPVI CMR, with an average RV telediastolic volume of 154.0 ± 27.1 ml/m^2^. Finally, 4/21 patients (19.0%) had preoperative EPS with negative programmed ventricular stimulation.

**Table 2:** Electrocardiographic and echocardiographic parameters prior and following PPVI

	Before	Short term	Medium term	Last visit
Electrocardiogram, ms (SD)				
Mean PR interval	166 (± 37)	162 (± 27)	160 (± 29)	165 (± 36)
Mean QRS duration	155 (± 28)	156 (± 26)	154 (± 28)	158 (± 22)
Mean corrected QT interval	438 (± 46)	436 (± 37)	430 (± 39)	446 (± 27)
Transthoracic echocardiogram				
Mean sub-pulmonary ventricle systolic peak pressure to PA gradient, mmHg (SD)	40.2 (± 19.5)	28.8 (± 15.4) ^†^	29.1 (± 11.9) ^†^	31.7 (± 14.8)
Mean sub-pulmonary ventricle systolic peak pressure to RA gradient, mmHg (SD)	46.0 (± 13.7)	38.8 (± 10.9) ^†^	37.2 (± 11.5) ^†^	40.6 (± 12.6) ^†^
Mean LVEF, % (SD)	57.92 (± 12.70)	61.98 (± 6.84)	62.42 (± 10.12)	59.07 (± 7.28)

*PA* pulmonary artery, *PPVI* percutaneous pulmonary valve implantation, *RA* right atrium

^†^*p* < 0.05 when compared with before PPVI

### Percutaneous Pulmonary Valve Implantation

A total of 22 procedures were performed on 21 patients. The two most common indications for PPVI were significant pulmonary valve insufficiency (PI) (*n* = 10, 47.6%) and mixed regurgitation/stenosis (*n* = 10, 47.6%). Isolated pulmonary stenosis was a rare indication (*n* = 1, 4.8%). Implantation characteristics are presented in Table 3. A total of 16 Melody® valves (76.2%), 3 Edwards Sapien® valves (14.3%) and 2 Venus P-valves® (9.5%) were implanted. Sub-pulmonary outflow tract pre-stenting before PPVI was performed in all but two patients implanted with a Venus P-valve®. No major or minor VA occurred during the procedure.

**Table 3:** Percutaneous pulmonary valve implantation data

Number of patients/number of interventions	21/22
Type of valve, *n* (%)	
Melody®	16 (76.2)
Edwards Sapien®	3 (14.3)
Venus P-Valve®	2 (9.5)
Landing zone, *n* (%)	
Stented conduit	15 (71.4)
Stented native RV outflow tract	4 (19.1)
Native RV outflow tract	2 (9.5)^†^
Non pre-stented conduit	0
Mean minimal landing zone diameter, mm (SD)	
Pre-implant	19.6 (± 3.5)
Post-implant	20.4 (± 4.6)
Mean sub-pulmonary systolic peak pressure, mmHg (SD)	
Pre-implant	51 (± 16)
Post-implant	39 (± 8)
Mean sub-pulmonary systolic peak pressure to BP ratio, % (SD)	
Pre-implant	57.7 (± 19.3)
Post-implant	40.1 (± 11.9)
Mean pulmonary capillary wedge pressure, mmHg (SD)	10 (± 2)
Pre-implant PI grade, *n* (%)	
Free	14 (66.6)
Severe	5 (23.8)
Moderate	1 (4.8)
Mild	1 (4.8)
None	0
Post-implant PI grade, *n* (%)	
Free	0
Severe	0
Moderate	0
Mild	10 (47.6)
None	11 (52.4)

*RV* right ventricle, *BP* blood pressure, *PI* pulmonary insufficiency

^†^Both were implanted with a Venus P-valve®

### Follow-Up

#### Primary Outcome

Patients were followed for a median of 29.6 months (IQR 10.9–44.0). Three patients (14.3%), all of whom had TOF or TOF-like physiology, experienced a major ventricular arrhythmic event (Table 4). Two patients were unable to have pre-implant CMR due to retained epicardial temporary pacing wire from previous surgery. It showed no other scaring than VSD patch closure in the third one. None of them have had pre-procedural EPS. No significant independent risk factor was found (Table 5).

**Table 4:** Summary of patients with severe arrhythmic events

#	Age at PPVI (years)	Age at event (years)	Sex	Congenital anatomic diagnosis	Sub-pulmonary outflow tract conduit type at PPVI	Primary implant indication (stenosis, regurgitation, mixed)	Valve (mm)	Event type
1	13.9	14.7	M	Double-outlet RV (Fallot type) with pulmonary valve atresia	Hancock® 18 mm	Mixed	Melody® (18)	Spontaneous sustained VT
2	13.4	16.1	M	TOF	Contegra® 16 mm	Regurgitation	Melody® (22)	Inducible sustained VT
3	14.6	15.6	M	TOF	Transannular patch	Mixed	Melody® (20)	Resuscitated cardiac arrest

*EPS* electrophysiology study, *PPVI* percutaneous pulmonary valve implantation, *TOF* Tetralogy of Fallot, *VT* Ventricular tachycardia

**Table 5:** Comparison between groups with and without severe ventricular arrhythmia with regards to established risk factors in TOF patients

	Patients without events (*n* = 18)	Patients with events (*n* = 3)
Median follow-up, months (IQR)	28.4 (9.5–53.5)	32.9 (26.8–32.9)
Median age at PPVI, years (IQR)	17.0 (14.7–18.3)	13.9 (13.7–14.3)
Median BMI at PPVI, kg/m^2^ (IQR)	19.3 (18.0–21.8)	21.2 (19.2–24.1)
TOF or TOF-like physiology, *n* (%)	12 (66.7)	3 (100.0)
Surgery before implantation		
Previous palliative shunt, *n* (%)	6 (33.3)	1 (33.3)
Transannular patch, *n* (%)	5 (27.8)	2 (66.7)
Median age at corrective surgery, months (IQR)	15.4 (9.2–26.1)	8.5 (8.4–9.3)
Baseline clinical symptoms		
Palpitations, *n* (%)	2 (11.1)	0
Syncope, *n*	0	0
Baseline ECG		
Mean QRS duration, ms (SD)	164 (± 25)	156 (± 6)
Mean QTc, ms (SD)	434 (± 46)	461 (± 42)
Baseline TTE		
Mean LVEF, % (SD)	59.04 (± 11.67)	50.65 (± 22.42)
Moderate or severe PI, *n* (%)	17 (94.4)	3 (100.0)
Moderate or severe tricuspid regurgitation, *n* (%)	4 (22.2)	2 (66.7)
Mean pulmonary capillary wedge pressure, mmHg (SD)	11 (± 2)	9 (± 2)

*PPVI* percutaneous pulmonary valve implantation, *BMI* body mass index, *TOF* Tetralogy of Fallot, *TTE* transthoracic echocardiogram, *LVEF* left ventricular ejection fraction, *PI* pulmonary insufficiency

The first patient had a double outlet right ventricle (DORV) with pulmonary valve atresia corrected with an 18 mm Hancock conduit after a Blalock–Taussig shunt. A Melody valve was implanted at the age of 13.9 years for severe conduit degeneration. It resulted in complete resolution of pulmonary insufficiency with moderate residual obstruction (peak systolic gradient of 48 mmHg at 3 months) due to patient-prosthesis mismatch. At the age of 14.7 years, 10 months post-PPVI, he presented with sustained monomorphic VT terminated with external electrical shock (Fig. 1B). An ICD was implanted and beta-blocker therapy initiated. After over 2 years of follow-up, he remained free of recurrent VT. The second patient had TOF and underwent corrective surgery with a transannular patch, followed by a Contegra conduit implant, and then PPVI at 13.4 years of age. Two and half years after, he presented with palpitations and presyncope. During EPS, fast sustained monomorphic VT (cycle length 200 ms) was induced during apical pacing at a drive train of 600 ms with three extra-stimuli and pace-terminated (Fig. 1C). Electroanatomical pace-mapping during sinus rhythm revealed a critical VT isthmus between the RV-to-PA conduit and VSD patch. Echocardiography revealed severe sub-valvular obstruction requiring surgical conduit replacement. The isthmus was transected by intraoperative cryoablation during the sPVR procedure, rending the patient non-inducible on postoperative repeat testing. He remained asymptomatic and without recurrent VT at 9 months of follow-up. A third patient with TOF who had surgical correction with a transannular patch, suffered a cardiac arrest 1 year post-PPVI at the age of 15.6 years. The first monitored rhythm was ventricular fibrillation. He was successfully resuscitated after receiving several external defibrillator shocks and underwent ICD implantation. He remained without recurrent VA after over 1 year of follow-up. None of the three patients had documented non-sustained VT on Holter monitoring or exercise testing prior to PPVI and all were implanted with a Melody® valve.

#### Secondary Outcomes

Two severe non-arrhythmic events related to the PPVI were reported, one significant pulmonary hemorrhage requiring admission to the intensive care (thus leading to a second procedure for PPVI), and one significant groin hemorrhage requiring surgical exploration immediately after PPVI. Both patients recovered without sequelae. Three patients experienced minor arrhythmic events during or right after PPVI. Two had minor VA, both following Venus P-valve® implantation. The first had frequent isolated PVCs, while the second had several episodes of NSVT. Both were discharged on beta-blocker therapy. Follow-up 24-h Holter monitors 2 months later showed resolution of the ectopy. The third patient had transient high grade atrioventricular block (with 2:1 ventricular conduction). On follow-up, 3 additional patients (14.3%) had 3 to 10-beat runs of NSVT on 24-h Holter monitoring treated with beta-blockers.

Other complications included a transient brachial plexus injury (*n* = 1; 4.8%), a femoral arteriovenous fistula (*n* = 1; 4.8%) treated conservatively, and a *Staphylococcus Aureus* endocarditis on a Melody® valve 1-year post PVVI (*n* = 1; 4.8%). Two patients (9.5%) subsequently went on to have repeated procedures. The first had LV dysfunction despite maximal medical therapy and received cardiac resynchronization therapy. The other had recurrent RV-to-PA conduit stenosis leading to sPVR and cryoablation (patient #2 in Table 4). Finally, one patient died of pulmonary hemorrhage at the age of 30 years.

## Discussion

Pulmonary valve replacement, be it surgical or percutaneous, prevents further RV dilatation and remodeling resulting from significant PI, which, in addition to ventricular scarring has been associated with an increased risk of long-term VA. However, the hypothesis that PVR alone decreases the risk for severe VA and sudden death has been largely refuted [17, 18] because it does not address the arrhythmogenic substrate directly. Some series, including mostly adult patients, have described the occurrence of minor VA following PPVI [12–14, 19]. To our knowledge, this is the first entirely pediatric study to specifically investigate the occurrence of severe VA post-PPVI. In our series of 21 pediatric patients with CHD and PPVI, severe VA occurred in 3/21 (14.3%) during a median follow-up of 29.6 months (IQR 10.9–44.0). Based on this experience, we have modified our institutional protocol to include programmed ventricular stimulation prior to PPVI in children (as is done for adults). In case of inducible VT, preoperative planning includes discussion between sPVR with simultaneous surgical cryoablation or PPVI associated with catheter VT ablation, considering local anatomy (myocardium thickness, calcifications and prosthetic material). It remains to be established which strategy will yield superior outcomes.

### Incidence of Severe VA

It is well established that the arrhythmia burden in adults with repaired TOF is increased compared to the general population, with a high prevalence of sustained atrial and ventricular tachyarrhythmias [20]. Khairy et al. [20] reported a prevalence of 14.6% (81/556 patients) in an adult cohort with TOF (mean age 36.8 years). Yet, severe VA may occur as early as 10–15 years post corrective surgery during the pediatric follow-up [21], as evidenced by the results of our study with severe VA occurring in patients ranging from 14.7 to 16.1 years of age. These results are consistent with a study by Beurskens et al. [22] that reported a VT prevalence of 6.3% (20/319) in a population with TOF ranging in age from 15 to 36 years and followed for 3.5 years. The prevalence in our study is likely higher due to the fact that all patients were referred for PVR and, hence, had important hemodynamic sequelae.

### Mechanism of VA

The transient occurrence of minor VA (frequent single PVCs and NSVT) in the 6 months post-procedure in 19.0% of patients corroborates the findings of previous series reporting an increase in the prevalence of benign VA in the post-PPVI implantation period [12–14]. These are thought to be secondary to contraction-excitation feedback induced by myocardial stretching during repeated ballooning and the placement of stents in the sub-pulmonary outflow tract. In addition, a certain degree of stent protrusion in the sub-pulmonary outflow tract can occur, particularly with the Venus P-valve® which has a flared shape at its proximal end [13, 19] (Fig. 1A). This arrhythmia burden usually subsides once the contact areas scar and become electrically silent [14]. There appears to be no significant increase in ventricular ectopy long term [18]. Moreover, when comparing PPVI to sPVR cohorts, the ventricular ectopy burden seems comparable in the medium and long term [23]. Regarding our patients with more severe VA, 2/3 had documented monomorphic VTs that were probably re-entrant, as well described in TOF [24]. In those two patients, it is unlikely that the approach of PPVI has affected the development of severe VA.

### Predictive Factors for Developing VA

Our study was underpowered to detect predictive factors for significant VA in children. Qualitatively, all three patients with VA had TOF-like physiology and free pulmonary regurgitation, two had transannular patches, and the QRS duration was no longer than the general cohort. The QRSd was not significantly different before and after PPVI, as previously reported [12, 17, 25]. Plymen et al. previously described shortening of QRS duration only in the subgroup of patients who had PPVI for pulmonary regurgitation [26]. Additional studies are required to determine whether high risk features identified in predominantly adult cohorts with TOF are applicable to children.

### Role of Programmed Ventricular Stimulation Prior to PVR

In patients with TOF, inducible VA by programmed ventricular stimulation has a high predictive value for future VT and sudden cardiac death in TOF [27, 28]. The delineation of anatomical isthmuses (i.e., corridors of slow conduction between electrically inert border) has allowed for better identification of potential targets for catheter ablation [24, 29, 30]. Although additional studies are required, there is some evidence that surgical cryoablation of identified critical isthmuses could be effective in rendering potential arrhythmogenic substrates non-inducible [17, 31]. In patients with clinical or inducible VA, PPVI could potentially cover the critical isthmus [32] and thereby impede access to myocardial tissue that is implicated in arrhythmogenesis. Multicenter studies are required to determine whether programmed ventricular stimulation should be routinely performed prior to PVR in all patients with TOF-like physiology, including children, to guide decisions regarding whether a percutaneous or surgical approach should be favored.

## Limitations

This is a single-center observational study with limitations inherent to the retrospective cohort design. Without a standardized prospective protocol, clinical assessment and ascertainment of outcomes could have varied over the 13-year period, reflecting the evolution of clinical care. The low number of CMR performed before PPVI (66.6%) was explained by limited availability of magnetic resonance imaging at the time of the first interventions and medical contraindications (ferromagnetic foreign material). The patient population had heterogeneous forms of congenital heart disease, with TOF-like physiology accounting for the majority. Finally, the use of three different valves impairs our ability to draw firm conclusions regarding the arrhythmic risk inherent to each valve. Consequently, a larger multicenter prospective study with a long-term follow-up is required to confirm our results and provide greater insight.

## Conclusions

In conclusion, severe VA can occur long after PPVI in a pediatric population, particularly in those with TOF-like physiology. Although the few events observed appear to be related to well-established substrates for VT in TOF rather than pro-arrhythmic effects from the percutaneous valves themselves, further studies are required to elucidate underlying mechanisms and identify associated factors. In light of these results, consideration should be given to risk stratification of children with TOF-like physiology with programmed ventricular stimulation prior to PPVI in order to identify potential substrates for sudden death and inform management decisions.

## Data Availability

The data that support the findings of this study are available on request from the corresponding author (MLB).
